# Morbidity and mortality in HIV-exposed under-five children in a rural Malawi setting: a cohort study

**DOI:** 10.7448/IAS.17.4.19696

**Published:** 2014-11-02

**Authors:** Oscar Divala, Charles Michelo, Bagrey Ngwira

**Affiliations:** 1 Department of Public Health, School of Medicine, University of Zambia, Lusaka, Zambia; 2 Department of Community Health, College of Medicine, University of Malawi, Blantyre, Malawi

## Abstract

**Introduction:**

Paediatric HIV infection significantly contributes to child morbidity and mortality in southern Africa. In Malawi as in most countries in the region, care of HIV-exposed children is constrained by the lack of area-specific information on their risk to dying and morbidity. This research estimates and compares morbidity and mortality events between HIV-exposed and -unexposed under-five children in a rural Malawian setting.

**Methods:**

Data for children under the age of five collected from January 2009 to June 2011 at a demographic and health site in Karonga district of northern Malawi were analyzed. Morbidity and mortality rates among HIV-exposed and -unexposed children were calculated and compared using Kaplan-Meier survival analysis and Cox proportional hazard regression.

**Results:**

Overall (n=7,929) cohort data of under-five children born in a demographic and health site represented 12380.8 person years of observation (PYO) of which 3.1% were contributed by HIV-exposed infants. Females accounted for half of the sample, and the overall mean age was 18.4 months (SD 13.4) with older children in the HIV–unexposed group. All-cause morbidity rate was 337.6/1000 PYO (95% CI 327.5/1000–348.0/1000) and HIV-exposed children morbidity rate was 1.34 times higher (p<0.001) compared to HIV-unexposed children. integrated management of childhood illness (IMCI) pneumonia was the most common diagnosis (39.3%) in this cohort. Child mortality rate was 16.6/1000 PYO (95% CI 14.5–19.1) from 206 deaths. HIV-exposed children had 4.5 times higher (p<0.001) mortality rate compared to the HIV-unexposed children. Higher mortality rates were observed in children under one year (129.2/1000 PYO) compared to older age groups.

**Conclusion:**

HIV exposure at birth has a greater impact on child morbidity and mortality especially in the first year of life. This underscores the need for targeted and synergetic interventions that included focused prevention of mother-to-child transmission (PMTCT) which could reduce HIV transmission to children in their infancy in this setting.

